# Proceedings of PAME: Second National Conference 2014

**DOI:** 10.12669/pjms.305.6056

**Published:** 2014

**Authors:** Shaukat Ali Jawaid

LAHORE: Pakistan Association of Medical Editors (PAME) organized its Second National Conference at University of Health Sciences Lahore from April 26^th^ to 27^th^ 2014 which attracted over three hundred fifty delegates and participants including two invited guest speakers from Iran i.e. Prof. M.B. Rokni Editor Iranian Journal of Public Health and Iranian Journal of Parasitology from Tehran University of Medical Sciences and Dr.Behrooz Astaneh Editor Iranian Journal of Medical Sciences from Shiraz University of Medical Sciences. The conference was organized in collaboration with University of Health Sciences (UHS), Eastern Mediterranean Association of Medical Editors (EMAME) and Health Research Advisory Board. Mr. Justice (Retd) Amer Raza Khan Chairman Punjab Healthcare Commission was the chief guest while an eminent educationist, former Principal of King Edward Medical College who is also former President of College of Physicians & Surgeons Pakistan Prof. Kh. Saadiq Husain was the Guest of Honour in the inaugural session. Prof. Mahmood Ali Malik another well known physician of the country and former Principal of King Edward Medical College graced the occasion with his presence as Chief Guest at the Conference Dinner which was also attended by many faculty members and eminent medical personalities.

**Inaugural Session**

Addressing the participants in the inaugural session **Mr. Justice Amer Raza Khan** said that Punjab Health Care Commission is an autonomous statutory body which is tasked with the responsibility of persuading and enforcing minimum service delivery standards of service. It will also protect them from excesses by the public functionaries and the beneficiaries of such services.

In order to evolve and induce the service providers, hospitals and clinics to become part of the effort to improve, the Commission has in consultation with the tertiary and secondary hospital managements, doctors , homeopaths , Tabibs and Nurses identified and laid down minimum standards of healthcare and initiated monitoring and evaluation of their working. It is for the first time that equal standards have been laid down for all public and private hospitals and clinics which will be monitored by an independent statutory agency and their performance evaluated. Unfortunately some private medical colleges and their affiliated hospitals have now contributed to the present dismal state of healthcare services in Pakistan. The situation is much worse in healthcare facilities located outside the major cities. As member of the Board of Governors of UHS, I have overseen its efforts in arresting the prevalent chaos in the field of medical education, he added.

Continuing Justice Amer Raza Khan said that Punjab Healthcare Commission has decided not to adopt an “enforcement mode” but to solicit and encourage compliance. Initial inspection have revealed a woeful state of affairs which should not be allowed to continue and now in a number of cases, discernible improvements have been achieved and this gives us hope that not only are we on the right track but the objective envisaged are achievable and will be achieved. It will take time and effort. The task is difficult because we are dealing with a profession and with investors who have of late become over assertive and at times defiant. Hence, a cautious approach is the need of the hour.

Yet another aspect of our responsibility, Justice Amer Raza Khan said was overseeing and implementation of ethical standards. We do not regulate the licensing of doctors which is done by the PM&DC which is also empowered to deal, with instances of malpractices. In fact PM&DC has jurisdiction over Teaching Hospitals and not all Hospitals. However, it does have disciplinary authority over medical practitioners in the provision of healthcare services by hospitals, clinics and medical practitioners and securing adherence to minimum healthcare standards mutually accepted by them all. Our complaint management system, Mr. Justice Amer Raza Khan further stated, is responsive and thorough to meet all the requirements of due process. Parties to a complaint are given adequate opportunities for presenting their cases. Specialists in relevant fields are engaged to give their opinion and investigatory visits to facilities complained against are conducted for collection of evidence. This Commission has the sole jurisdiction over cases pertaining to alleged medical negligence, malpractice or administrative failure and healthcare service providers have immunity against proceedings conducted against them at other forums. This has been an area which remained unattended for years. We see light at the end of the tunnel now and mutual efforts of all of us will surely not only ensure adherence to ethical values but also improve healthcare standards and resultantly restore and enhance the dignity of all involved in providing acceptable healthcare services, Mr. Justice Amer Raza Khan concluded.

**Prof. Kh. Saadiq Hussain** in his speech discussed in detail various aspects related to medical journalism i.e. Medical Research, the art of Medical Writing, Workshops, Peer Reviewing, Publication Ethics, role of the private sector and information technology. Scanning the conference programme, he said, shows that all relevant issues associated with medical writing and publishing have been covered in the conference for which the organizers need to be commended. Pakistan, Prof. Kh.Saadiq Hussain said has been a late starter in the field of medical journalism and there are genuine reasons for that. To find an answer to this one has to go back nearly seventy years. Medial Journalism is a tightly woven teamwork between authors, reviewers, editors and their teams, publishers, health professionals and policy makers. Any weak link at any point in this chain is enough to disrupt the whole process, he remarked.

Many people do not know that when Pakistan came into existence not a single link in the entire chain mentioned above was in place. We had only one medical college in the entire country i.e. KEMC which was severely depleted to the tune of about 70% by the departure/migration of non-Muslim teachers, doctors, administrators, nurses, laboratory staff and even the students. Those of us remaining were overwhelmed by millions of refugees, starving, sick, wounded, and shattered physically and emotionally. In this scenario the pre eminent issue was “Survival” and not ”Research”. There were no authors, hence no reviewers or editors. I as a senior medical student was working as a doctor, a nurse, a lab assistant, a ward bearer, a refugee camp worker ( as the need arose) and of course a student. This was the only human component, the material and financial component was even worse. However, slowly and steadily with faith in Allah and confidence in ourselves, hard work and dedication, the scenario began to change. More medical colleges started in major cities and the country initially with skeleton staff, makeshift or no campuses, and poorly equipped. However, gradually as most of us who had gone abroad usually UK, returned with MRCP or FRCS, and highly qualified faculty became available, the hospitals and colleges were adequately staffed and equipped. In those days Membership or Fellowship was the only exams. Soon after passing those exams, one became stem cell of the profession capable of becoming a member of the medical teaching faculty, a researcher, an author, a medical administrator or health policy maker even though one had received no training what so ever in any of these spheres. I had been teaching for twenty years before I attended the First WHO sponsored workshop on medical education at Lahore. With the passage of time, adequate number of faculty personnel, qualified teachers, numerous medical colleges, we had plenty of ”authors” and would be ”reviewers”. Although we used to have conferences and also wrote articles and case reports for the college journals, we had tremendous difficulty in access to reference materials. For this we often had to approach the British Council library. Eventually it was visionaries and pioneers like Mr. Ayaz Mahmood (Medical Newspaper) , Dr. Sarwar J. Zuberi and later many others including Major. Gen. Aslam, Mr. Shaukat Ali Jawaid and their colleagues who played a vital role in promoting medical journalism as an important subject in its own right.

Speaking about Research, Prof. Kh. Saadiq Hussain said that it was an undeniable fact that very little original research comes out of Pakistan in international journals, although research comprises the bulk of contents of journals. It is essential to identify the cause if we hope to correct the problem. Some of the reasons which Prof. Kh. Saadiq Hussain identified included lack of interest, lack of initiative or ability, lack of incentives, reward, lack of funding, inability to meet publishing criteria or all the above. Added to this was the lack of time due to complete involvement with the virus epidemic or rather pandemic CC which stands for Commercialization and Corruption. An in-depth study of the problem and its possible solution is the order of the day and must be tackled on an urgent basis, if we are to achieve our objective of promoting research and documentation.

Medical writing especially research articles have a specific format. It needs to be learnt and practiced to be accepted for publication. He suggested that training in medical writing should begin at undergraduate level to promote the “culture of documentation and research.” It is heartening to know that medical writing is already being practiced at some undergraduate medical institutions and in universities in Karachi and Peshawar. At the postgraduate level it is already mandatory but needs considerable improvement as about 50% of manuscripts do not satisfy the publication criteria. Research should have relevance to national issues and needs so that even policy makers can make use of results of medical research.

Workshops are an innovation in teaching technology which is extremely useful in converting passive listeners into active participants. Indeed they learn a lot but workshops are the means to an end not an end in themselves. Speaking in a lighter vein, Prof. Kh.Saadiq Hussain remarked that workshop is often treated like any other shop in town, when the shop closes, it is work as usual. He laid emphasis on getting feed back as to the results of such workshops. We conducted workshops for ten years regularly in King Edward Medical College but there was hardly any unit other than mine where simple changes like making and following check lists and OSCE and interactive lectures were routinely adopted in the bedside and ward teaching as well as lecture theaters. I even failed to convince Board of Studies in Punjab University to introduce only 25% MCQs in our final MBBS exams. He stressed the fact that all participants of any workshops must report back after a predetermined period of time in what way have they benefitted by the workshop in their routine work. Passing on the benefit to others, in this way we would be able to evaluate our own workshops usefulness.

Talking about Peer Reviewing Prof. Kh. Saadiq Hussain referred to the presentation by Dr. Jamshed Akhtar at the First National Conference on Medial Editing held in 2007 at Army Medical College wherein disappointing response of the reviewers was listed. It also emphasized the care in appointing reviewers to the extent that “we are still unclear what peer review actually does, and can its omission bring any change”. We need more workshops to train reviewers. Continuing Prof.Kh.Saadiq Hussain said that there are some universally accepted rules and regulations in medical publishing. Likewise in all professions there are black sheep who do not abide by the rules. The Committee on Publication Ethics (COPE UK) has laid down a code of conduct for authors, editors and publishers. It has listed as many as forty seven misdemeanors which can be committed and must be prevented and strictly penalized. The need to uphold professional ethics is much more even today than it ever has been, even institutions are not immune.

The mushroom growth of medical colleges will not make any significant impact on publications in the near future because most of the staff in these colleges is retired faculty of public sector institutions. We have been talking of poor writing habits in the past and if people did not write while in active service in youth, they are not likely to start after retirement. Information technology is here to stay and now it is “E everything” including e publications. It is about time that all undergraduate institutions include computer knowledge as an essential part of their curriculum. He concluded his speech by modifying a quotation from a distinguished medical editor Prof. Harvey reported by Mr.Shaukat Ali Jawaid Managing Editor of Pakistan Journal of Medical Sciences in one of his book “In general, the only people who love editors are their wives, husbands, children and parents” and Prof. Kh.Saadiq Hussain added to this list honest, dedicated, academicians.

**Dr. Maqbool H. Jafary** President of EMAME also briefly spoke on this occasion. **Mr. Shaukat Ali Jawaid** Secretary General of EMAME made a presentation highlighting the historical background of formation of EMAME and its activities, accomplishments so far besides showing glimpses from the First National Conference on Medical Editing held at Army Medical College Rawalpindi in 2007 as well as the EMMJ5 organized at CPSP Camps in Karachi in 2010. He also gave details of training courses organized by EMAME in collaboration with PAME at Karachi, Rawalpindi-Islamabad, Peshawar and Lahore besides workshops on medical writing at National University of Malaysia, Isfahan University of Medical Sciences as well as International conference on Medical Writing held at Dubai. **Dr.Akhtar Sherin** President PAME highlighted the activities of PAME during the last two years. He also announced that HEC has agreed to provide TURNITIN software for screening of manuscripts for plagiarism to journals who are members of PAME for one year to begin with.

Earlier **Prof. Maj. Gen. Muhammad Aslam** Vice Chancellor of UHS in his welcome address suggested that PAME should organize its biennial conference by rotation in all the provincial capitals of Pakistan, AJK, Gilgit and Baltistan as well as Federal Capital area. He offered the UHS Campus to organize WAME conference if it can be materialized. Pakistan, he further suggested, should take lead in establishing South Asian Association of Medical Editors (SAAMJE). He thanked chief guest, guest of honour for gracing the occasion with their presence. 

**Managing Affairs of a Medical Journal**

The first scientific session during the conference was devoted to a seminar on Managing Affairs of a Medical Journal. It was chaired by Prof. M. Akbar Chaudhry along with Dr. Fatema Jawad while Dr. Jamshed Akhtar acted as the moderator. **Mr. Shaukat Ali Jawaid** from Pakistan Journal of Medical Sciences was the first speaker who talked about how to run a successful biomedical journal. He pointed out that pressure to publish by the faculty to meet academic requirements and decision by various specialty organizations, medical institutions to have their own publications has resulted in more and more biomedical journals being launched by these institutions. However, they have to face lot o difficulties due to lack of any training or experience among those who are entrusted these responsibilities. Some of the essential requirements for running a successful journal, he said, are Good quality manuscripts, Minimum essential staff, Financial sustainability, Active Editorial Board, Good Quality of Reviewers, Increased visibility, Indexation in various databases, Online availability and Easy to use Website. To begin with always assess your strength and resources, move slowly and ensure steady progress, decide about the frequency whether it has to be Biannual, Quarterly, and Monthly. For many it will be better to start with biannual publication (2 issues in a Year) after obtaining the declaration. Carefully select the Editorial Board and Founder members, don’t go for Big Names but Good Workers for editorial board. In the early phase the contents in an issue could include Editorial or Editorial Note, One original article, One Review Article, One Case Report, Clinical Updates, Short Communication, News, Conference Reports, Obituaries, Quiz etc. Make sure that the print issue as well as the website of the journal has detailed comprehensive Instructions for Authors and publication policy, Letter of Undertaking regarding exclusive submission, scanning for plagiarism and approval from the Ethics Committee/IRBs before submission to the journal.

Online publication is economical, offers increased visibility and readership. It will attract more manuscripts but Print edition is essential. Prepare essential mailing list which should include libraries, institution, PM&DC, HEC and prospective authors. Prepare Reviewers Data Base, have minimum essential staff and ensure timely communication with authors as well as reviewers. Once the journal has been recognized by various bodies and covered by important databases, it will attract more submissions. Follow author friendly policy, seek help and assistance from colleagues, thank the reviewers and impress upon the editorial board members to play an active role. Ensure regular timely publication and give importance to capacity building of staff by attending training courses, workshops. Once you have attracted enough manuscripts, start concentrating on quality, seek coverage by PubMed or PubMed Central, Reuter Thompson/ISI which is known for Impact Factor. Finally it is also essential to know your Rights and Responsibilities as an Editor and continue concerted efforts to further improve the quality of the contents and standard of the journal as the financial and human resource permit.

**Dr. Bilal Mirza** highlighted the success story of APSP Journal of Case Reports (AJCR) which is an electronic open access journal and official publication of the Association of Paediatric Surgeons of Pakistan (APSP). This was a joint venture undertaken by Dr. Bilal Mirza along with Dr. Jamshed Akhtar. The decision to launch this journal, he said, was taken in March 2010. Within six months, the maiden issue of the journal was brought online on 14^th^ August 2010, with a slogan of “a portal for research, innovation, and knowledge”. We became member of Directory of Open Access Journals (DOAJ), within 3 months which greatly improved the journal’s visibility. At the start, the correspondence between authors, reviewers and editors was through the email. The manuscript submission was also allowed only through email. We followed double blind peer review system. We applied for the PubMed Central (PMC) indexing in year 2011 and it was approved in year 2012.That was a big day for the “two men army”. The journal now attracts manuscripts from many countries. We are using open journal system for manuscript submission, tracking, review, and publication. Future plans include induction of the young blood in the editorial board to facilitate journal’s workflow. Our motto is “When there is a will there is a way without compromising on integrity, standards, and ethical principles”, he added.

**Dr. Saeeda Baig** talked about their experience of launching an E journal from Ziauddin Medial University, Karachi. **Mr. Muhammad Zafaruddin **from JCPSP in his presentation pointed out that the main goal of their journal was to promote and disseminate clinical and evidence based medical research as well as to educate and promote research culture and medical writing skill. He described success story of JCPSP spanning 23 years of its existence. The journal was launched in 1991 as a quarterly journal which became bimonthly in 1995, monthly in 1999 and included in Index Medicus (MEDLINE) in 2002. It is also indexed by more than a dozen leading indexing agencies of the world.

JCPSP was the first biomedical journal in Pakistan to attain Impact Factor in 2009; the journal has a dedicated website with free access, no fee is charged from contributors, and it is a trends setter in many areas with its own printing press and the full support of internationally recognized body – CPSSP. Managing Editor is the key person who organizes all the activities, dealing with managerial and administrative issues and is responsible for communication with contributors, reviewers, sister journals and editorial board members.

JCPSP has an organized office set up, a full time Managing Editor, two honorary Editors, two honorary Associate Editors and full time office staff including an Editorial Assistant, a Graphic Designer, two Statisticians, a Bibliographer, a Publication Officer, three Publication Assistants and an office boy, he remarked.

**Prof. Nasir Khokhar** Editor Rawal Medical Journal from Islamabad talked about challenges the Medical Editors have to face in Pakistan. Though Medical editors have various challenges everywhere in the world but in Pakistan, he said, we have unique problems which may not be considered elsewhere by medical editors. We start with extremely poor submissions. The authors do not read and follow the “Instructions for authors” which can be different for different journals. Several authors do not know the proper use of electronic submission process. Multiple times the manuscripts have to be returned for re-submission. The manuscripts are often much disorganized. The usual format is not followed. The concepts are not clear. Frequent repetitions make the manuscript lengthy and redundant. Authors do not revise according to reviewers’ suggestions. Reviewers delay the review a lot. Several of them have no specific review expertise while many are unfamiliar with web-based review, he added.

**Second Session**

Dr. Behrooz Astaneh from Shiraz Iran along with Prof. Khalid Masood Gondal from CPSP chaired this session which was moderated by Dr. Muhammad Irfan.

**Sarwar Zuberi Memorial Lecture**

**Prof. M.B. Rokni** Editor of Iranian Journal of Public Health from Tehran University of Medical Sciences delivered Dr. Sarwar Zuberi Memorial lecture. The theme of his presentation was the present situation of medical journalism in Iran. He pointed out that as Iran has prepared a 20-years perspective document which aims that the number of scientific journals should increase so that Iran can get the 1^st^ rank in the region in terms of science production. To achieve this objective, lot of emphasis is being laid on not only increasing the number of journals but also improving their quality and standard. At present there are 315 approved journals by the Iranian Ministry of Health Overall, 154 journals are published in English language followed by 161 in Persian with English Abstract.

Tehran University of Medical Sciences, Prof. Rokni said with 31 journals published in English is the leader followed by Shiraz and Mashhad universities, which publish 20 and 17 biomedical journals respectively. In terms of indexing in authentic databases, there are 66 journals present in ISI master list, from which 23 medical journals are covered by Web of Sciences which have got an Impact Factor. The rate of IF varies from 0.086 to 1.2. As for SCOPUS, 87 from 127 journals are considered medical. Bedsides, 49 journals are covered by PMC/PubMed/Medline.

 According to data released by Thomson Reuters ISI, in 2013, the number of published papers in this database, was 32600 with Iran ranked 2^nd^ after Turkey with 38000 papers in the region .Based on SCIMAGO data, during the period 1996-2012, Iran in the arena of Medicine is allocated the following items: H index: 82; Documents: 38147; Citable documents 35492; Citations 131537; Self-citations 43207 and Citation per document 3, 45. Prof. Rokni further stated that every year the Iranian MOH based on different indices such as Technical indices, covering by 1-3 degree scientific databases, timing, circulation etc., classifies all Medical Journals. The criteria are >95 points: Level 1; 65-94: Level 2 and <95 Level 3. Accordingly the International Journal of Preventive Medicine from Isfahan University of Medical Sciences with 120 points had the highest rank followed by the least ranked journals (three cases) each of which of 30 points. A birds’ eye view on the current situation of Medical Journalism in Iran shows a satisfactory situation based on international criteria but obviously the quality also needs to be improved along with the quantity, he concluded.

**Dr. Saba Sohail** from JCPSP made a presentation on editing qualitative studies in medical education for a general biomedical journal. Her conclusions were that the main editing issue was the author-editor in-house disagreement regarding study design and format as qualitative studies differed markedly from quantitative studies which form the majority of submissions and citations. We have now prepared separate guidelines for qualitative studies which will be included in the revised manuscript submissions, she added.

**Dr. Fatema Jawad** from JPMA was the next speaker who described how they handled frequent submissions from a single author at JPMA. She pointed out that a single author submitted to them 46 articles, 29 original articles and 17 letters, from February to October, 2012. The articles were on diverse subjects ranging from Dermatology to medicine, paediatrics, surgery and microbiology. The corresponding author was a student for all articles and first author for 8 originals and 12 letters. The co-authors were senior faculty members. The average submissions were five per month. This gave a cause for suspicion as how could a student get time to conduct research, analyze the results and write the articles at this frequency, besides performing his clinical work and studies?

 The required documents were checked and it was observed that the signatures of all authors on the submission certificate appeared forged and the authors of all articles had an email on yahoo.com. Guidelines of Committee on Publication Ethics were followed and letters were sent to the author with relevant questions. The author was questioned, who wrote back “I am one of the best researchers of my country and have multiple publications in every field of medicine and have won multiple prizes.”The Vice-Chancellor of the university was contacted, with no response. The chief of the research committee of the university was contacted who supported the author in a very unconvincing manner. However we had no satisfactory reply from the author. The case was put up in the COPE Forum in June 2013 and the members advised to ask the university to depute someone to talk to the student, Secondly the co-authors should be contacted for their view and some higher authority or ministry of research should be taken into confidence on the matter. It was also suggested that the concerned author could be a psychopath .Two co-authors could be contacted who said this was a case of fraud. They had not co-authored the articles and did not know the first author. An internet search revealed the retraction of a published article by the same author due to authorship issues. Another senior co-author wrote back that he was shocked to see the paper published. He had no knowledge about it. He wrote that he did not know this author and never met him. He also claimed that his published data was stolen. He said he would forward this message to the ethics department and complain to the university.

The case was followed up in the COPE Forum of September, 2013 the members advised to contact a higher authority of the University for investigating the case. If investigations were not performed then editor should publish the findings of the journal’s enquiry. They also advised to contact other journals with articles of the concerned author. The correspondence was carried out with the Secretary National Ethics Committee who wrote that the university was conducting an investigation and confirmed that more misconduct had been detected against this author. He also suggested that the journal should take an independent decision on the unprocessed articles lying in the journal’s office The decision of the editorial board was to close all the 27 files on grounds of Fraud and debar the author. The Secretary National Research Ethics Committee of the author’s university and COPE Forum were informed. It was concluded that this was a case of fraud and was detected by vigilance. COPE guidelines and advice of the Forum members were very helpful, she concluded.

**Dr. Jamshed Akhtar’s** presentation was on study designs and statistical analysis- Editor’s dilemma. He pointed out that most of the editors are not formally trained in epidemiology and biostatistics. Deficiencies in study designs and statistics nullify purpose of publication. Many journals do not have dedicated staff to address this part of the manuscripts. We tried to find out that deficiencies in study deign and statistical analysis in the manuscripts submitted to JCPSP. It was a retrospective analysis. Total number of manuscripts included in the study was sixty. Statistical tests were found incorrect in thirty one; descriptive statistics was incorrect or incomplete in another thirty-five while inferential statistics was incorrect in thirty nine manuscripts. Most of these manuscripts had to be revised numerous times. He laid emphasis on training the principal investigators in this area. He was of the view that editors must have background knowledge, attend short courses while PAME should also organize some workshops to train the editors, he added.

**Online Learning Resource for Editors**

Pakistan Association of Medical Editors formally launched its Online Learning Resource for Editors during the Second National Conference. It is the brain child of **Dr. Masood Jawaid** Assistant Editor at Pakistan Journal of Medical Sciences who is also its Project Director. This learning resource was formally commissioned in the second scientific session on Day one of the conference where Dr. Masood Jawaid also gave a brief demonstration about the contents it contains. e-Resources for Editors: Free stuff **by Dr. Masood Jawaid (Pak J Med Sci),** Study designs for quantitative study by **Dr. Jamshed Akhtar** (J Coll Physicians Surg Pak),Authorship **by Shaukat Ali Jawaid**(Pakistan Journal of Medical Sciences), Holistic approach for Epidemiological Study Designs with emphasis on RCT by **Dr. Farwa Rizvi**(Islamabad Medical and Dental College). It offers free access to the editors and authors of biomedical journals on PAME website. www.pame.org.pk

**Prof. Umar Ali Khan**, Pro Vice Chancellor, Isra University made a presentation on quality medical writing. He stated that an author of a medical manuscript must be clear about the types of writing like internal reports, regulatory reports, Conference presentations; oral and poster presentations and Journal articles. Journal articles include original research paper, review article, case report, letter to editor etc. As it is said if you fail to plan then you Plan to fail so be realist for original research by designing a clear research question, have statistician’s opinion about the study design, work with an open mind, authorship should be clearly decided. Maintain patient confidentiality. Author can have guidance from International Committee of Medical Journal Editors uniform requirements for manuscripts submitted to biomedical journals. The manuscript should be kept simple and to the point. The data must be accurate free of false results and plagiarism. Even negative results should also be presented. The message of research, he stated, should be clearly reflected in introduction, methods, results and discussion. The conclusion drawn should be logical. Whenever we get the review of our article we should not get mad rather we should feel happy that reviewers and editors are helping us to improve the quality of our publication.We must experience medical writing as it is said ***“****There is no way to get experience except through experience” *he remarked.

**Workshops**

 In the afternoon of Day One of the conference i.e. April 26^th ^2014, four workshops were organized. The workshop cum interactive seminar on Medical Writing was conducted by Mr. Shaukat Ali Jawaid and Prof Anwar Siddiqui. Dr. Fatema Jawad along with Dr Behrooz Astaneh from Shiraz Iran and Dr. Muhammad Irfan conducted the workshop on Publication Ethics. Prof. M.B.Rokni from Tehran, Iran was the facilitator in the workshop on Journal Indexing while Dr. Masood Jawaid along with Dr. Akhtar Sherin conducted the workshop on Electronic Publishing.

**Third Session**

On Day Two of the conference April 27^th^, this session was chaired by Prof. M.B.Rokni from Tehran Iran along with Prof. Junaid Sarfaraz Khan Pro VC UHS Lahore. **Dr. Masood Jawaid** was the first speaker who in his presentation discussed the progress made by Pakistan Journal of Medical Sciences during the Year 2013.Pakistan Journal of Medical Sciences, he said was one of the leading biomedical peer reviewed medical journal from Pakistan which has to its credit tremendous achievements during the last year. These include bimonthly frequency from quarterly, Digital Object Identifier (DOI) assignment for every manuscript with an agreement with CrossRef. For detection of plagiarism, use of iThenticate software by CrossCheck screening every manuscript accepted for peer review. PubMed Central (PMC) indexing is another milestone with availability of all of its manuscript in XML format effective January 2013. In addition in collaboration with ‘The Medical Writers’ the Journal has helped training of more than 100 doctors within the country and overseas through its ‘Online Research Course’. He also discussed how PJMS achieved these goals and gave some suggestions for other Journals of the region how they can achieve all this.

**Dr. Jamshed Akhtar** from JCPSP talked about analysis of articles based upon qualitative study designs: Are the Editors doing justice? Qualitative research, he pointed out includes broad category of partially overlapping research types including action research, case study research or grounded theory etc. He also highlighted the differences between qualitative and quantitative research besides discussing the three qualitative methods. His conclusions were that editors should prepare themselves for yet another challenge. They must understand qualitative designs so as to do justice while processing these manuscripts.

**Shaukat Ali Jawaid** talked about citation amnesia. This presentation was based on a case study. He pointed out that Pakistan Journal of Medical Sciences in its June 2013 issue published a Review on Depression covering inflammatory Hypothesis. This manuscript had 89 references mostly covering the Reviews published in the last ten years. This prompted a European Psychiatrist himself Editor of a Psychiatric Journal to call it an intellectual property theft and citation plagiarism. He complained that his work has not been properly cited and wished that the paper should be withdrawn and an apology from the authors published by the journal. He even threatened to take the case to COPE and report to other agencies like Higher Education Commission in Pakistan, head of the institution of the authors, besides various international databases. He also gave deadlines for the response from the Editor. He was asked to send his viewpoint which will be published along with the response from authors but he insisted that the manuscript must be removed from the website and he be given four to five pages to respond to this failing which, he again threatened to take up the issue with COPE and other bodies. Such a behaviour and threatening tone in his communications raised many ethical questions related to publication ethics, duties and responsibilities of Editors as well as their rights and privileges. Dr. Behrooz Astaneh COPE Council member who is also affiliated with Pakistan Journal of Medical Sciences as an Ombudsman was consulted who opined that we had adopted a correct approach. Correspondence with this author through e mail continued for over six weeks and when he insisted on removal of the manuscript from the website, he was told that it was not possible and we are fully aware of our rights and responsibilities as Editors and it worked. The take home message in this presentation was that while dealing with such cases of citation amnesia or plagiarism don’t get emotional, be polite in language you use. Keep all the record of such correspondence. Ask for Advice from senior colleagues. Be mindful of your Responsibilities, give chance to readers to comment, criticize on published contents. Publish all this with response from the authors. Safeguard and protect your Rights & Privileges and refuse to be intimidated and don’t succumb to pressure tactics and such threatening e mails and dictatorial attitude. **Dr. Saeeda Baig** from Karachi talked about tribulations of reviewing a journal manuscript. 

**Ramsha Zaheer **from Karachi talked about Editorial Problems in Students' Articles Submitted to JPMA**. **The objective of this retrospective study was to document the editorial problems in articles submitted to the student’s corner at JPMA from January 2012 to December 2013.The problems were divided under five headings. As far as the quality of the manuscript was concerned, the most common problems were faced in statistics, where either the frequency or percentage would be missing, and the references, where 50% did not adhere to the correct format. Multiple submissions as letters to the editor were also noted (45%; n=67)   which did not add anything new which was not already available in textbooks. Lot of delays were faced in submitting an ethical review board approval or a similar document (42.4%; n=14, for 2013) after JPMA made it compulsory to submit a document verifying the approval instead of just mentioning it in the paper. Further delays in the processing of the article were made due to late response from the peer reviewers (48.5%; n=16 for 2013) and the authors, who would understandably be busy with their exam schedules. However, the most important issue faced was that the authorship criteria were not followed. These included supervisor being the first author (2), changes made in the author order (2), deleting authors from the initial submission list (1) and making submission to the journal without informing the co-authors (1). Her conclusions were that it is important to guide students not only how to write a scientific paper but also ethics regarding research. 

**Dr. Osama Ishtaq**, Project Manager PakMediNet made a presentation on audit of medical journals of Pakistan. PakMediNet, he said was an index database of articles published in the medical journals of Pakistan. It is the only database available online and is providing free services for the last 13 years. Users can search using various variables which includes free phrase search, search on titles, journal names, author names, abstracts and keywords. Currently this database contains 75 medical journals of Pakistan comprising of more than 21,000 articles (titles and abstracts) and 1530 issues. 

**Prof. Mohammad Aslam**, Prof. of Obstetrics and Gynaecology from Lahore talked about advantages, disadvantages and alternatives to Peer Review. He pointed out that peer review is the assessment by an expert of the scientific material submitted for publication. It is considered as gold standard. However, despite its widespread use and costs, little hard evidence exists that per review improves the quality of published biomedical research. Peer Review does ensure that published research is original, important and timely, technically reliable, consistent and well presented. It is a quality control measure but its weakens is that it is slow, expensive, wastes lot of academic time, it is highly subjective, prone to bias, can be easily abused and is considered very poor at detecting gross defects and academic fraud. However, it does improve quality of the manuscripts before publication. Post publication reviews, manuscript management and peer review software, citation analysis and impact factor were mentioned as some of the alternatives to peer review. Open peer review is the latest. Peer review system, certainly needs some improvements to make it more useful, he added.

**Mehwish Hussain **from DUHS discussed the Statistical Flaws in Manuscripts Submitted to JPMA.Non compliance with statistical rules leads toward likelihood for rejection of manuscript. The objective of this study was to find out the frequent flaws observed in submitted manuscript during last two years. The most recurrent problem was incorrect or unreported sample size calculation of the study. Alignment of the statistical analysis with objective and title of the study was observed to be the foremost flaw. Specification of related sampling technique and explicit sampling procedure were also absent especially in randomized controlled trial and community based studies. Description of external validity of sampled population was also missing. Inferential statistics were applied and reported without testing the prior related assumptions. Inaccurate advanced statistical tests was observed as the key flaw in this regard. Her conclusions were that statistical inaccuracies ranged from sampling stage to advanced data analysis in manuscript submitted to JPMA. Medical researchers must involve statistician not only for data analysis but at the initial stage of the study.

**Fourth Session**

Prof. Nasir Khokhar along with Prof. Noshin Wasim Yousuf chaired this session while Prof. Ijaz Hussain acted as the moderator. 

**Manzar Saleem Memorial Lecture**

**Dr. Behrooz Astaneh**, from Iranian Journal of Medical Sciences who is also Council Member of COPE delivered Prof. Manzar Saleem Memorial Lecture. Title of his presentation was COPE- the aims and new features. He discussed at length the issues related to authorship disputes, conflict of interest, duplicate submissions, the redundant publications, plagiarism, data fabrication and falsification. The reason for unethical behaviour, he opined, could be intentional i.e. pressure to publish or due to lack of knowledge. Many researchers do not know what can be considered as misconduct while many editorial board members are not aware of the exact definition of various misconducts and many editors do not know how to tackle these misconducts. The objective of various bodies like COPE, EMAME, WAME and PAME are to treat lack of knowledge cause of ethical misbehavior among editors and researchers. However, he remarked that even training won’t do anything to those who deliberately commit ethical misconduct. He then highlighted various famous cases of fraud.

The objective of COPE, Dr.Behrooz Astneh said was to promote integrity in research publications. At present COPE has over nine thousand members from 75 countries and it covers almost all academic disciplines and fields. COPE provides advice and resources to editors and publishers on all aspects of publication ethics. It has a twenty member council from eleven countries. COPE has so far produced Code of Conduct and Best Practice Guide for Journal Editors, Code of Conduct for Journal Publishers, Guidelines for Retracting articles. It has produced Flow Charts on redundant, duplicate publications, plagiarism, fabricated data, changes in authorship, Ghost, guest or gift authorship, conflict of interest, general suspected ethical concerns and reviewer’s misconduct. COPE membership offers many advantages including free participation in COPE seminars, access to eLearning packages and use of ethical audit tools. More recently COPE has produced some eLearning programmes aimed at improving the editor’s abilities to deal with publication misconduct, to give editor’s deeper understanding of publication ethics besides how to detect and handle misconduct. COPE publishes a monthly Digest and organizes regional seminars in different countries, he added.

**Shaukat Ali Jawaid** discussed Burnout Syndrome (BOS) among medical journal editors. Defining BOS, he said it was a work related disorder. It consists of symptoms of emotional exhaustion, physical fatigue, and cognitive weariness which results from ineffective coping with enduring stress. It is also associated with decreased job performance, low career satisfaction and affects behavior, personality as well as professional goals. Some of the causative factors include little appreciation of the work, little time for the family, little time for relaxation and holidays. Burnout Syndrome is a well known entity and has been extensively documented among various categories of healthcare professionals like psychiatrists, critical care physicians, nurses, those working in ICU, CCUs, oncologists, those looking after terminally ill patients, medical residents, those working in Emergency Medicine Departments and primary healthcare physicians. 

Medical Editors also suffer from burnout syndrome but we tend to ignore it. It has not been documented in the literature as well. He then gave the results of a small study which was conducted among members of Pakistan Association of Medical Editors (PAME).Out of the fourteen responses; two were Full Time and twelve part time Editors. Eight out of fourteen Editors reported to have noticed Burnout Syndrome. Their most frustration came from authors and reviewers. Full time professional Editors working at small journals with financial, human manpower constraints but aspiring for indexation in major data bases, eager to improve the standard and quality of their journal, establishing high expectations, those totally committed and devoted are at a greater risk of suffering from burnout syndrome as they try to achieve their objective with little or no support. He then suggested some of the positive interventions to overcome this problem of Burnout Syndrome like Make a Team and Delegate responsibilities, have written contract with rights and responsibilities, Insist on EC/IRB approvals of studies which is helpful in taking care of Scientific misconduct, academic fraud, Screening for plagiarism of all manuscripts, Training of Journal support staff and giving them Incentives to Retain them. Continuing, he further stated that it is nice to be an Idealist but Be a Realist- have realistic expectations, Go Slow and have modest Goals to begin with, Attend Workshops, Training courses, go for continuing professional development, Share thoughts with colleagues and learn how they tackle such problems you are faced with which will provide lot of Energy to cope with such a situation.

**Prof. Anwar Siddiqui** from AKUH was the next speaker who highlighted the need to have better coordination between institutions and journals to ensure integrity of research and publications. Plagiarism, Falsification, wrong attribution, misleading information was mentioned as some of the misconducts. He then referred to a case wherein a second year medical student got his paper published in a Korean Journal without the knowledge of supervising faculty, the work was sponsored by a granting agency that was also not acknowledged. The institution when came to know about it requested the editors to put the publication on hold till few things were sorted out. In another case samples for the study came from UAE, they were analyzed in Pakistan and France but none of the authors were from UAE. No ethical approval was obtained from anywhere and it was published in Pakistan. There are reports of fake or misleading research institutes hence in case of misconduct who should be held responsible, he asked? Prof. Anwar Siddiqui suggested that biomedical journal editors need to follow the COPE guidelines, carefully check the author’s affiliations, nature of job and insist on Ethics Committee/IRB approval before accepting any manuscript for publication. Both Journals and Institutions have important duties. Journals are responsible for the conduct of their editors for safeguarding research record and ensuring reliability. Institutions should have research integrity officers and inform journals of proven misconduct, provide information if asked for by the journals which can go a long way in checking authorship disputes and misleading reporting. For better coordination institutions should develop policies on authorship, misconduct and intellectual property rights. 

**Prof. Ijaz Hussain, **Editor, Journal of Pakistan Association of Dermatologists spoke about the problems and challenges faced while managing their journal. My 11-year experience as Editor of this Journal, he said, had been a turbulent voyage. Problem faced included scarcity of funds, lack of quality peer-review, ethical issues like plagiarism, misappropriation of authorship, duplicate submissions, and urgent publication. The future challenges that we are facing include getting the journal impact factor, indexation in the MEDLINE, recognition by Higher Education Commission of Pakistan and changes in website. 

**Prof. Muhammad Moin **Editor Pakistan Journal of Ophthalmology talked about the impact of maintaining a website for the Journal. He gave the results of a survey conducted among the readers of their journal and received twenty one responses. It showed that the contents of the journal have improved over the last few years; the topics covered in the journal are relevant. There were ten questions in all in this survey covering various aspects. Many felt that peer review was convenient using computer editing and e mail and retrieval of favorite articles was also easier in electronic version. . His conclusions were that maintaining the website of the journal improves the satisfaction of the readers.

Dr. S.H. Waqar from PIMS Islamabad presented comparative analysis of articles published in national and international medical journals. Quality of the medical journal, he said, was directly related to the competence and interest of authors, reviewers and the editors. In this study they assessed one hundred fifty two articles which included 104 international and forty eight national. For comparison, various sections of manuscripts like abstract, introduction, methods, results and discussion besides conclusions and references were reviewed. Study design was also looked into. Among the international manuscripts, 87.5% were excellent as compared to 23% in national manuscripts. Again 12.5% were good in international manuscripts as compared to 6% in the national manuscripts and 52% of the national manuscripts were found to be highly unsatisfactory. The study conclusions were that quality of national articles is highly variable. Many articles do not follow the recommended tools of systematic methods and there was need for continuous training of authors, reviewers as well as editors.

**Figure:**
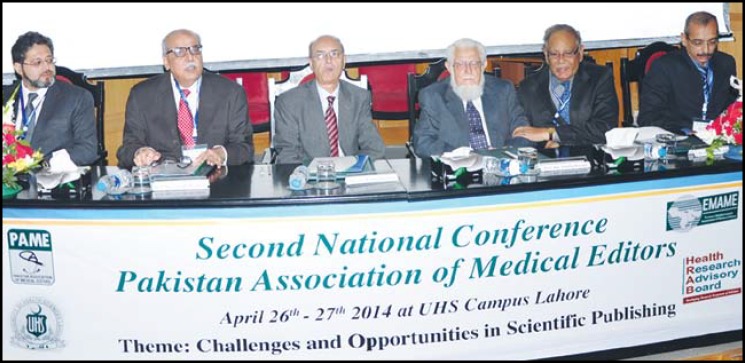
*Justice Amer Raza Khan (Chief Guest) along with Prof. Kh. Saadiq Husain (Guest of Honour) chairing the inaugural session. Also sitting on the dais from (L to R) are Dr.Akhtar Sherin President PAME, Prof. Maj. Gen.Muhammad Aslam VC UHS, Dr. Maqbool H. Jafary President EMAME and Dr. Jamshed Akhtar General Secretary PAME*

**Figure:**
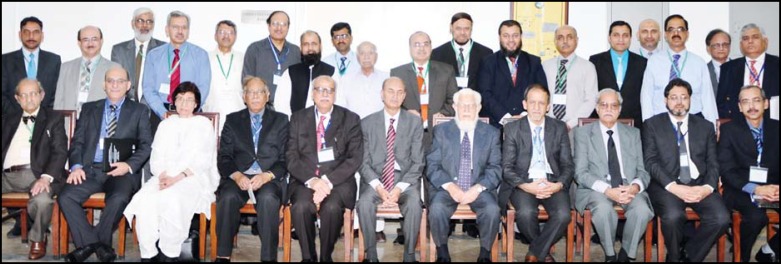
*Members of the organizing committee and some senior faculty members of UHS photographed along with Chief Guest Justice Amer Raza Khan and Guest of Honour Prof. Kh. Saadiq Husain after the inaugural session.*

**Figure:**
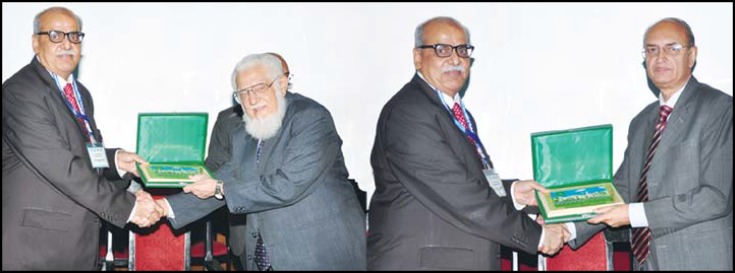
*Prof. M. Akbar Chaudhry (extreme right) along with Dr. Fatema Jawad and Dr. Jamshed Akhtar chairing the first scientific session *

**Figure:**
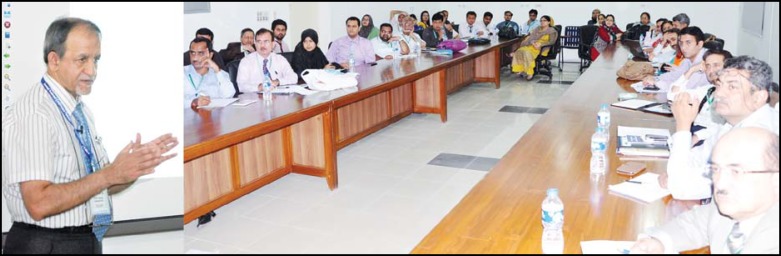
*Dr. Behrooz Astaneh from Shiraz Iran along with Prof. Khalid Masood Gondal from CPSP and Dr. Muhammad Irfan chairing the second scientific session.*

**Figure:**
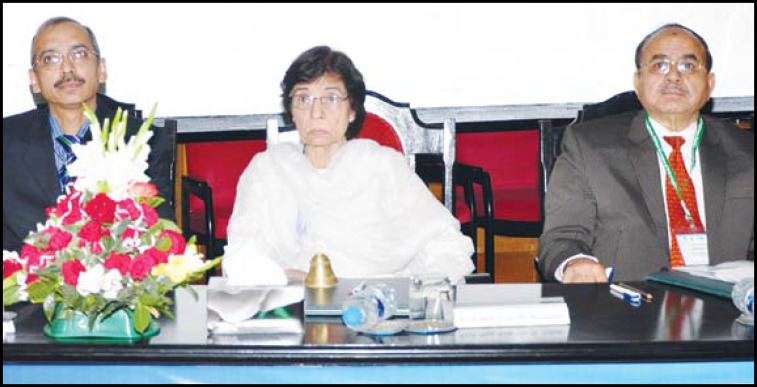
*Prof. M.B.Rokni from Tehran Iran (R) along with Prof. Junaid Sarfraz Khan Pro VC UHS Lahore chairing the third scientific session.*

**Figure:**
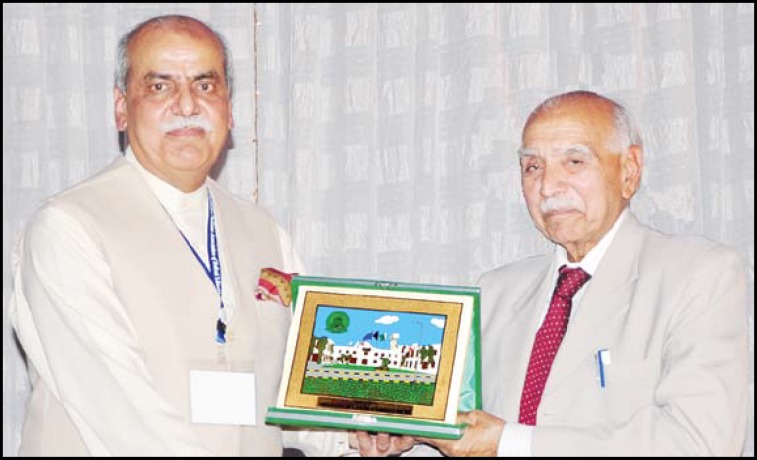
*Prof. Nasir Khokhar ( Right) along with Prof. Noshin Wasim Yousuf chairing the fourth scientific session.*

**Figure:**
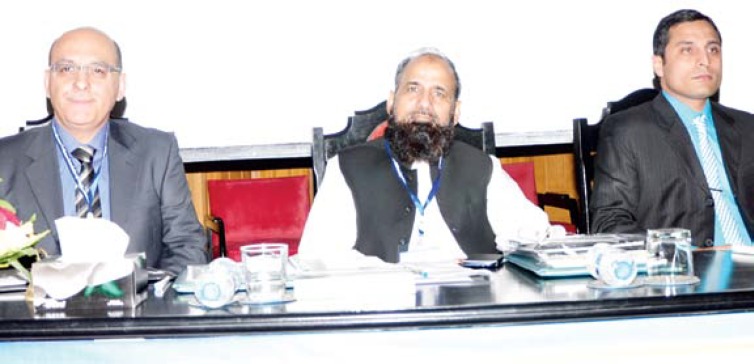
*Prof. Maj. Gen. Muhammad Aslam VC UHS presenting mementoes to Prof. Kh. Saadiq Husain and Mr. Justice Amer Raza Khan during the inaugural session.*

**Figure:**
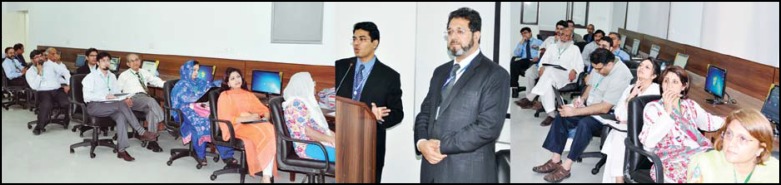
*Prof. M. B. Rokni Editor Iranin Journal of Public Health from Tehran University of Medical Sciences Iran conducting the workshop on Journal Indexing*

**Figure:**
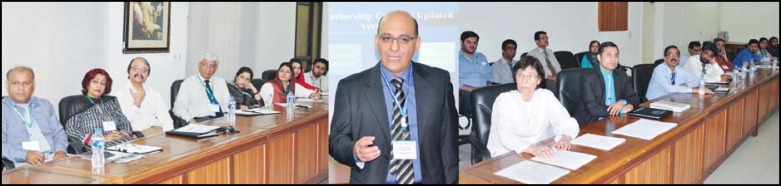
*Dr. Behrooz Astaneh Editor Iranian Journal of Medical Sciences from Shiraz University of Medical Sciences Iran facilitating the workshop on Publication Ethics. Other facilitators in this workshop included Dr. Fatema Jawad Chief Editor JPMA and Dr. Muhammad Irfan Editor JPMI.*

**Figure:**
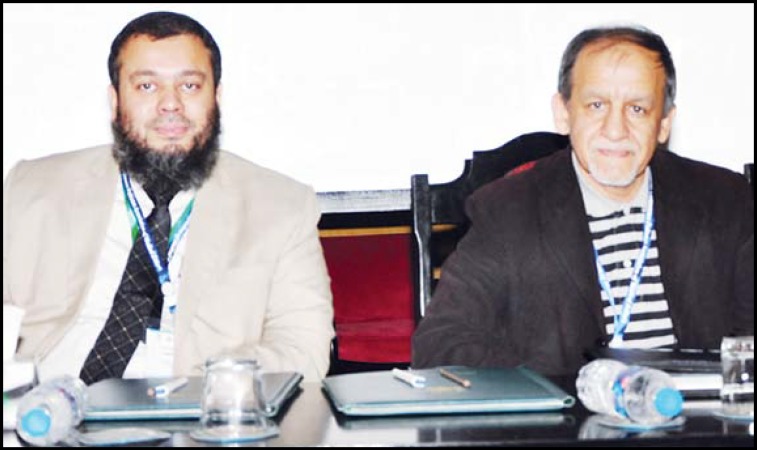
*Dr. Akhtar Sherin President PAME chairing the* *General* *Body Meeting on April 26* ^th^ *, 2014. Also sitting on the dais from (L to R) Dr. Jamshed Akhtar, Dr. Fatima Jawad, Prof. Maj. Gen. M. Aslam and Prof. Nasir Khokhar.*

**Figure:**
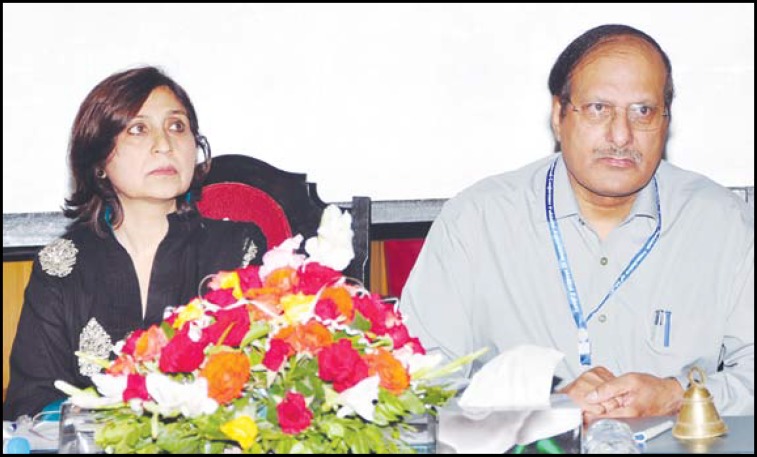
*Prof. Maj. Gen. M. Aslam VC UHS presenting a mementoe to Prof. Mahmood Ali Malik who was the Chief Guest at the Conference Dinner. *

**Figure:**
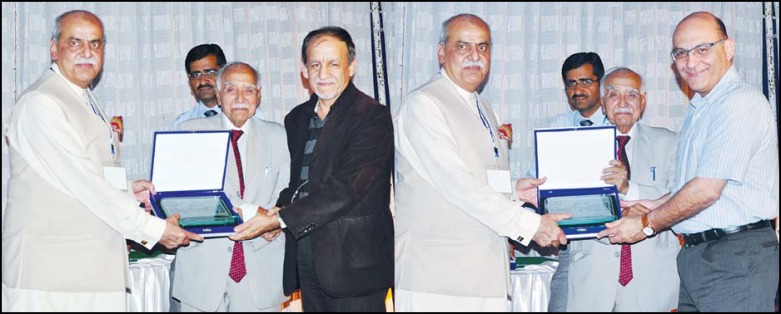
*All rights of Biomedica have now been acquired by University of Health Sciences after completing the legal formalities. It will henceforth be the official journal of UHS. It was formally launched at the PAME Second National Conference Dinner on April 26th 2014. Picture taken on the occasion shows Prof. A. H. Nagi and Prof. I. A. Naveed formally presenting the copies of Biomedica to Prof. Maj. Gen. M. Aslam and Prof. Junaid Sarfraz Khan VC and Pro VC of UHS respectively.*

**Figure:**
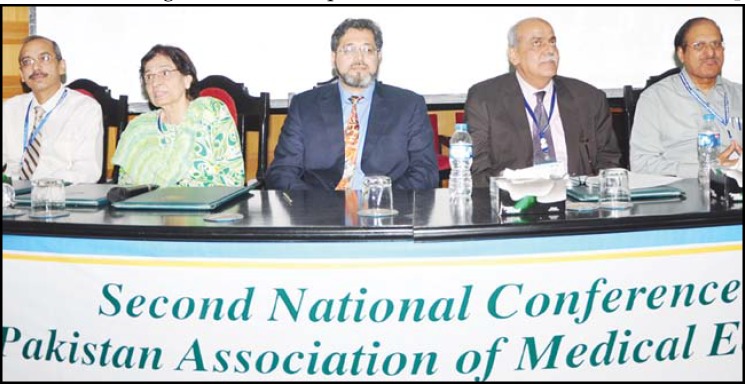
*Prof. Mahmood Ali Malik along with Prof. M. Aslam VC UHS presenting mementoes to Prof. M. B. Rokni and Dr. Behrooz Astaneh the two invited guest speakers at the PAME Second National Conference at the conference Dinner on April 26th 2014.*

**Figure:**
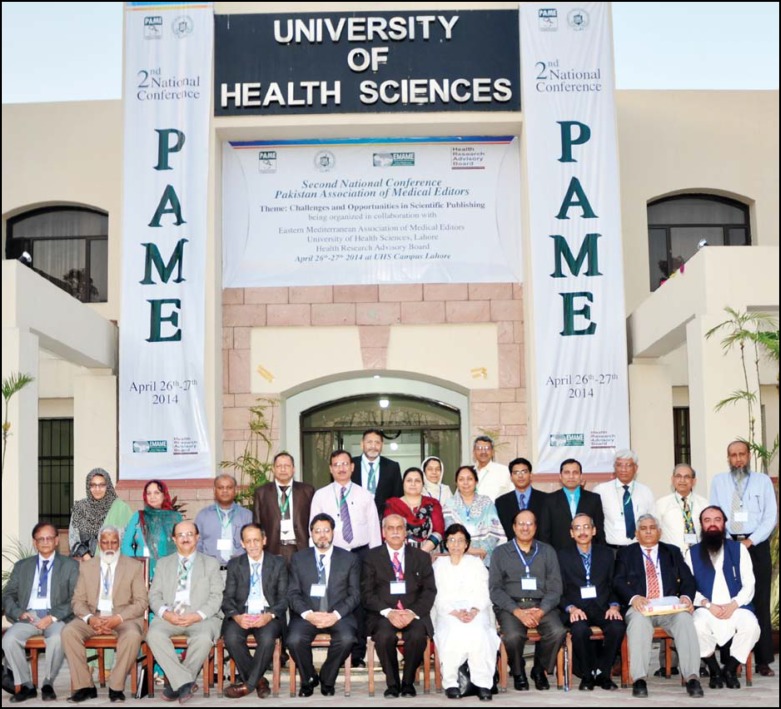
*Group photograph taken after the General Body Meeting shows PAME Executive members along with some other participants.*

**Figure:**
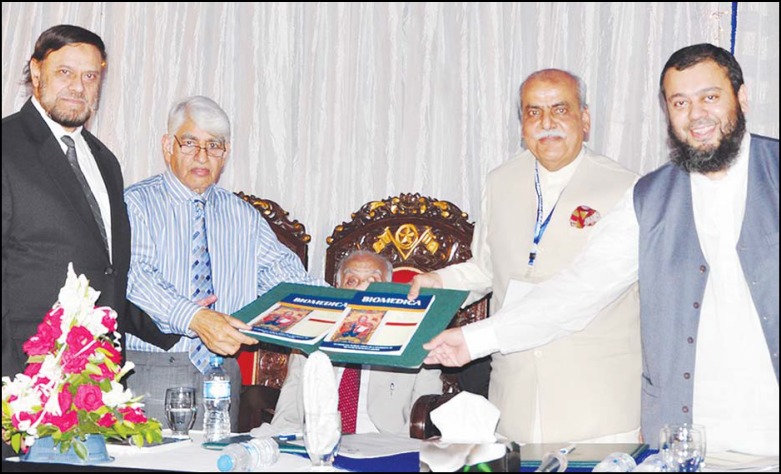
*Dr. Masood Jawaid Assistant Editor Pakistan Journal of Medical Sciences along with Dr. Akhtar Sherin President PAME conducting the workshop on Electronic Publishing.*

Prof. Nazeer Khan from DUHS spoke about the pattern of dental research in Pakistani dental journals during the last decade. He pointed out that only two dental journals are being published regularly in Pakistan. Two hundred forty three articles were published in Journal of Pakistan Dental Association and 338 were published in PODJ. Most of the published papers were original articles 73.7% followed by Case Reports 1 3.4%. About one third of these articles belonged to epidemiology followed by patient’s series while manuscripts on clinical trials and dental materials were only 5% each. Two third of the published articles could be described as clinical research while one third consisted of basic research and oral pathology and community dentistry. Among the clinical research articles, papers related to orthodontics accounted for 34.4%.

**Dr. Muhammad Irfan** from JPMI Peshawar talked about mutual cooperation among journals: Is it effective in preventing publication misconduct? He highlighted the benefits of mutual cooperation of Journal of postgraduate Medical Institute and Khyber Medical University Journal in preventing issues pertaining to publication misconduct. He was of the view that although every editor wants to have a smooth sailing regarding publication of his journal, it is only possible in Utopia and one has to face incidents of Publication misconduct. So, if these issues do occur, one can either try to avoid these issues or be ready to tackle them. He then shared three cases describing the ways used to tackle such issues by mutual cooperation. He suggested that a mechanism needs to be devised between the journals to avoid such incidences. This can be done in the form of sharing the cue sheets, before publishing the article or discussing such cases at PAME Listserve and other forums or/ and educating authors, reviewers and Editors.

**Conference Recommendations**

 In the General Body meeting held in the evening of First Day of the Conference i.e. April 26^th^, Dr. Akhtar Sherin the outgoing President of PAME presented the following recommendations of the conference which were all approved after discussion.

1. PAME should be given representation in journal’s indexation committees of PMDC & HEC.
2. Institutions/ universities should arrange/encourage CPD programs in medical journalism and research in collaboration with PAME
3. Institutions/ universities should establish IRBB to address ethical issues in research and In future, IRBB approval may be considered mandatory for articles to be published in medical journals
4. All journals should follow the COPE guidelines in addressing issues related to publication ethics
5. Biennial conference of PAME should be held in all provinces on rotation basis, with subsequent workshops in different areas of Pakistan
6. Pakistan should take a lead in establishing SAAME (SAARC ASSOCIASTION OF MEDICAL EDITORS) and Medical journals from Pakistan should ensure maximum participation in the next EMAME Conference likely to be held in Iran.

